# Death of Professor Hard

**Published:** 1851-12

**Authors:** 


*Death of Professor Hard.* — The last No. of the Western Medico-Chirurgical Journal, announces the death of Dr. Nicholas Hard, Prof. of Anatomy in the College of Physicians and Surgeons of the University of Iowa. We quote from the Journal just named, the following tribute to the memory of the deceased:

"Professor HARD, although comparatively a young man, occupied a distinguished position in the ranks of the profession of the West. He was an indefatigable student, a successful practitioner, an able and accomplished lecturer, and eminently adorned the profession by his great moral worth, and the purity of his private and professional character. Few, indeed, exhibited his zeal in the cultivation of the profession, of which he was one of the brightest ornaments."

